# Nutrition, Immune Function, and Infectious Disease in Military Personnel: A Narrative Review

**DOI:** 10.3390/nu15234999

**Published:** 2023-12-02

**Authors:** Adrienne Hatch-McChesney, Tracey J. Smith

**Affiliations:** Military Nutrition Division, U.S. Army Research Institute of Environmental Medicine, 10 General Greene Ave, Natick, MA 01760, USA; adrienne.m.mcchesney.civ@health.mil

**Keywords:** infection, obesity, energy intake, micronutrients, respiratory tract infections

## Abstract

Consuming a diet that meets energy demands and provides essential nutrients promotes a healthy immune system, while both under- and over-nutrition have been associated with immune dysfunction. Military personnel comprise a unique population who frequently endure multi-stressor environments, predisposing them to immune decrements. Additionally, 49% and 22% of active duty U.S. military personnel are classified as overweight and obese, respectively. A literature search on PubMed was conducted to identify studies, reports, review papers, and references within those sources relevant to the topic area. Military personnel experiencing either under- or over-nutrition can suffer from degraded health, readiness, and performance. Insufficient intake of nutrients during military operations increases infection risk and negatively impacts infection recovery. Energy, protein, iron, zinc, and vitamins C and D are nutritional areas of concern that may impact immune competence in a multi-stressor environment. Over-nutrition can promote accretion of excess body fat and obesity, which contributes to a chronic inflammatory state that coincides with immune impairments. Prioritizing efforts to optimize nutrient intake is one approach for reducing disease burden and improving readiness. This review discusses nutritional concerns concomitant to multi-stressor environments that impact immune function, and the relevance of obesity to infectious disease risk in the military population.

## 1. Introduction

Development and maintenance of a healthy immune function are essential in order to avoid detrimental pathophysiological processes, such as allergies and chronic inflammatory responses [1]. Immune function depends on the combined actions of the innate and adaptive immune systems, working collectively in the prevention and recovery from infections. The non-specific innate immune system acts by recognizing and initiating a quick response against invading pathogens. The innate immune system is comprised of physical barriers (i.e., skin, epithelial lining), biochemical barriers (i.e., mucus, gastric acid), antimicrobial peptides, and a non-specific leukocyte-mediated cellular response (i.e., neutrophils, macrophages, natural killer cells) that defends against pathogens, typically through inflammatory processes [2,3]. The adaptive immune system responds by eliciting a targeted response to specific invading organisms [4]. The antigen-specific adaptive response is mediated by B and T lymphocytes that kill virally-infected cells and produce antibodies targeted to destroy specific pathogens [2,3]. The adaptive immune system can generate immunological memory, enabling a rapid antigen-specific response upon another infection by the same pathogen; however, the adaptive response to a pathogen takes several days or weeks to develop [2,5]. Whether an infection occurs depends on the integrity of the host immune system and its synergistic network of cells, cytokines, lymphoid organs, humoral factors, and the pathogenicity of the invading organism [5]. The interdependent cellular and barrier immune network relies on energy and nutrients to develop and maintain protection against disease, infection, and adverse physiological reactions [2,6]. Furthermore, the immune system works in conjunction with other systems, such as the digestive system, where micronutrients and gut microbiota are linked to inflammatory and immunomodulatory mechanisms [7,8]. Gut microbiota regulate the mucosal immune system, which includes airway epithelia that protect against respiratory infection [9].

A variety of biological factors (e.g., age, body fat, dietary intake, sleep deprivation, psychological stress) influence immune competence [3,10,11,12,13,14]. Maintaining immune competence among military personnel is particularly important, as acute respiratory infections are problematic in training environments and can account for up to 27,000 lost training days and 3000 hospital-bed days annually for recruits [15]. In 2021, respiratory infection resulted in 8466 total bed days for hospitalized military personnel [16]. Susceptibility to respiratory infection increases in response to military-relevant stressors during periods of high physical activity, increased training intensity, and inadequate sleep [9,17].

The relationship between nutrient status and susceptibility to infection is relevant to the military population, which is at risk for deficiencies of immune-enhancing micronutrients when training in or deployed to austere environments [18,19,20]. During military operations, warfighters often face conditions that promote the spread of pathogens (i.e., crowded living spaces), and periods of prolonged exercise, sleep deprivation, and psychological stress combined with inadequate energy intake, thus increasing infection risk under these circumstances [21,22]. Stress hormones, as well as humoral and cellular immune responses (i.e., the two main mechanisms of immunity with the adaptive immune system) to military training, have been linked to vulnerability for stomach illness [23]. During deployment, infection from novel pathogens to which an individual is immunologically naïve raises concern about the transport and spread of disease to outside populations or community members [24]. In addition, the emergence of the highly contagious SARS-CoV-2 (COVID-19) virus has threatened the health, readiness, and lethality of military personnel [25,26,27]. The term ‘lethality’ is used instead of mortality to reflect the military context, referring to the capability and capacity to destroy [28]. Rapid spread of the virus in close living quarters and the impact of short sleep duration on hospitalization rates from COVID-19 have been observed in military populations [29,30].

Changes in adipose tissue in response to overnutrition additionally contribute to infection susceptibility due to chronic low-grade inflammation associated with changes in cytokine and hormone signaling [31]. Trends in the prevalence of overweight and obesity have been rising among military personnel in the U.S [16,32] and elsewhere [33,34]. Consequential behaviors from occupational stress may be related to overweight and obesity among military personnel, compromising readiness for duty [33,35]. This narrative review will consider the impact of stressors relevant to military occupational demands on nutrition-related factors that modulate immune responses. 

## 2. Methods

A PubMed database search was performed in October 2021, and an updated search was performed in March 2023. The search terms used were “military and immune function”, “nutrition and immune function”, “stress and immune function”, “obesity and immune function”, and “obesity and military”. The search criteria specified literature from within the past six years of the search date and identified studies, reports, review papers, and references within those sources relevant to the topic area. Due to the limited number of studies that examined immune function in the military-relevant population, we considered all papers, regardless of how immune function was assessed. For topic areas in which not enough recent literature was found, the search was extended to include prior years. Published data from military laboratories and organizations within the military community were additionally consulted, and search criteria was extended to the past 25 years due to the limited number of available military-relevant studies. Additionally, all environmental scenarios were included when examining available research. This includes references that measured immune function during training scenarios, deployment (combat and non-combat zones), and in the laboratory under controlled conditions.

## 3. Summary of Review Results and Related Discussion

### 3.1. Energy, Protein, and Immune Health

**Figure 1:**
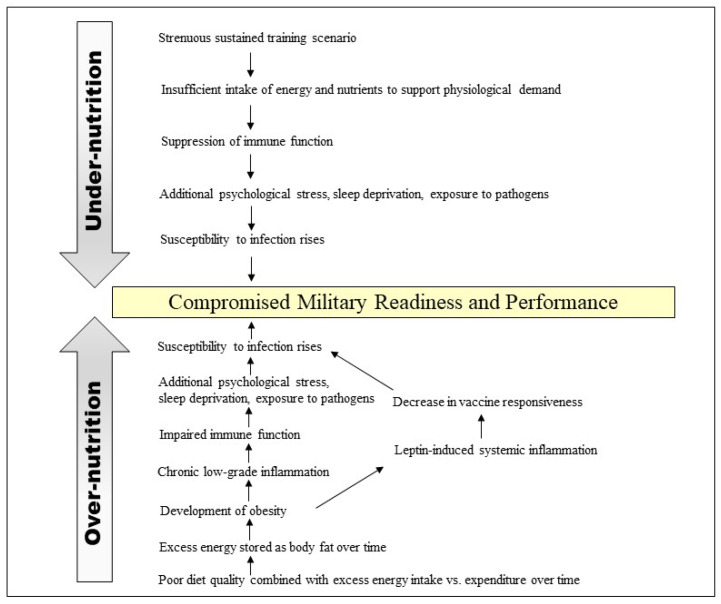
Immune-related consequences of under- and over-nutrition on military readiness and performance.

Inadequate dietary intake of energy and/or protein has a profound effect on innate and adaptive immune function [36,37]. Immune cells use a variety of biochemical pathways to obtain the energy and metabolites necessary to sustain their physiological demands [37]. In the case of malnutrition, the lack of nutritional signaling can impact immune cell proliferation, metabolism, and subsequent differentiation [31]. During infection, the immune system requires approximately 25–30% of calories expended by basal metabolism [37]. Thus, the immune function of individuals consuming inadequate dietary energy is likely to be compromised, increasing their susceptibility to infection [38]. The consequences of inadequate energy intake on immune function have been observed in both civilian and military populations [39,40,41]. For example, elite physique athletes in a low-energy availability state from energy deficit/increased exercise that resulted in 13% weight loss exhibited alterations in immune function parameters indicating immunosuppression compared to control athletes [41]. These alterations included dysregulated hematopoiesis, suppressed immune cell proliferation, diminished systemic inflammation, and decreased antibody and chemokine secretion [41]. The physical demands of military training (e.g., combat field exercises, repeatedly carrying heavy loads), combined with energy restriction as a result of logistical constraints or training objectives, especially puts trainees at risk for low energy availability [21]. This, in turn, may lead to suppressed immune function and susceptibility to infection (Figure 1). Correlations between the magnitude of energy deficit during rigorous military training programs with the degree of suppression of mucosal or cellular immune function and concurrent infection have been reported [42,43]. For example, U.S. Soldiers, who were attending the very strenuous Special Forces Assessment and Selection School experienced negative energy balance and ~4% weight loss over a 19-day training period, also exhibited impaired immune function [40]. This was evidenced by lymphocyte changes in the blood and delayed-type skin hypersensitivity responses [40]. However, a subset of those Soldiers who received a multi-nutrient beverage with vitamins/minerals twice daily demonstrated attenuated immune decrements compared to others who received a placebo drink [40]. Similarly, U.K. military personnel undergoing eight weeks of strenuous training while provided with a daily multi-nutrient food supplement experienced reduced energy deficit and body mass loss compared to those who did not receive the supplement [39]. Furthermore, decreases in immune biomarkers [circulating leukocytes, lymphocytes, monocytes, salivary secretory immunoglobulin A (SIgA) excretion] observed in the non-supplemented group were prevented in the supplemented group. This observed modulation is favorable for immune function integrity, and in particular, the increased salivary SIgA rate could potentially reduce respiratory infection risk [39]. 

Protein status is linked to cell-mediated immunity and vaccination response [44]. In cases of severe protein deficiency, such as protein-energy malnutrition often seen in developing countries, immune function is impaired, with reduced epithelial and physiological barrier function, and degraded function of macrophages, neutrophils, and natural killer (NK) cell activity [36]. Additionally, atrophy of lymphoid organs and T lymphocyte deficiency is observed with dietary protein deficiency, which in turn raises susceptibility to viral and bacterial pathogens and opportunistic infections [45]. Military operational stressors (e.g., sleep restriction and psychological stress) impair immune function [46,47] and suppress the inflammatory response and essential pro-inflammatory cytokine production during the early phases of wound healing [48,49]. However, protein intake above the low end of the military Dietary Reference Intakes (mDRI) [50] may speed healing time when immune function is impaired by those stressors [51]. A placebo-controlled, parallel-design study of healthy individuals experiencing acute sleep restriction observed delayed skin barrier recovery following the induction of an experimental wound, and study participants who received a nutrient beverage providing supplemental protein, amino acids, and other nutrients during the wound healing period showed potential benefits in the local immune response compared to participants receiving a placebo [52]. A follow-on randomized, crossover study administered the same supplemental beverage during acute sleep restriction and found that participants’ skin barrier was restored ~1.2 days faster compared to when they consumed a placebo beverage and standard protein diet [51]. There is evidence that supplementing protein after exercise sessions in Marines Corps recruits undergoing basic training could be linked to fewer healthcare visits [53]. Moreover, in elite athletes, high protein intake during strenuous training has been associated with restored (e.g., to normal-intensity training load) leukocyte distribution, and fewer self-reported symptoms of upper respiratory infection were observed during periods of high vs. normal protein intake under normal training conditions [54]. 

### 3.2. Micronutrients of Concern

Consuming a diet with inadequate energy also increases the risk of consuming inadequate amounts of micronutrients. Micronutrients have a role in mediating inflammatory responses and modulating chronic and inflammatory autoimmune diseases [55]. Suboptimal micronutrient status increases the risk of acquiring infectious diseases, and the subsequent duration of the infection [2,56]. Micronutrient deficiency and periods of sustained undernutrition can blunt cytokine responses and immune cell trafficking responses to pathogens [7]. Dysregulation of immune homeostasis may occur during conditions of deficiency of certain trace elements such as zinc and copper due to the negative impact on the number and function of immune cells [55]. Micronutrients also have the potential to modulate chronic and inflammatory autoimmune diseases via other mechanisms. For example, oral supplementation with vitamin B2 resulted in lower serum levels of inflammatory markers, higher plasma free thiols, and symptom improvement in patients with Crohn’s disease [55,57]. Vitamin A may modulate autoimmune and inflammatory disease symptoms through such mechanisms as downregulating the expression and signaling of Toll-like receptors (TLR), enhancement of T cell migration to inflamed tissues, and induction of cell differentiation [55].

Micronutrient status can influence a viral pathogen’s genetic makeup, thereby contributing to the development of new pathogens. For example, selenium in the form of the amino acid, selenocysteine, forms part of the catalytic site of peroxidases, which have a role in antioxidant defense, redox signaling, and redox homeostasis during viral infection [55,58]. Because viral pathogens generate oxidative stress, selenoproteins are critical to host defense. In the event of selenium deficiency, selenoprotein expression decreases, facilitating increased oxidative stress, which can lead to viral genome mutations, increasing viral severity and pathogenicity [58]. Selenium deficiency has been associated with increased pathogenicity of several viruses (i.e., hepatitis B, hepatitis C, influenza), and some evidence indicates that supplementation may reverse or reduce risk for other pathologies associated with oxidative stress, such as cancer, neurogenerative, cardiovascular, and infectious diseases [58].

A robust evidence base supports the role of vitamins C and D, iron, and zinc in supporting immune function. Vitamin D regulates mineral metabolism and skeletal health but also has immunomodulatory effects on cells of the innate and adaptive immune systems through endocrine, paracrine, and intracrine mechanisms [59]. The majority of immune cells possess the vitamin D receptor and its activating enzyme, 1-α-hydroxylase, and vitamin D has been shown to support the physical barrier (cells lining epithelial surfaces such as the skin, respiratory tract, gastrointestinal tract) defense against pathogens, reduce inflammation during infection, and stimulate the production of antimicrobial compounds [36,60,61]. In its active hormonal form, vitamin D supports the physical barrier through upregulating mRNA production of the antimicrobial peptide, cathelicidin, to promote the clearance of bacteria at the barrier sites in epithelial cells [61]. In human macrophages, when vitamin D status is adequate, upregulation of the expression of the vitamin D receptor and 1-α-hydroxylase activates vitamin D during pathogen invasion [61]. This, in turn, upregulates cathelicidin expression, which has a role in the innate immune response to bacterial infection [61]. Deficiency has been associated with autoimmunity and higher sensitivity to infection by microbial pathogens [62]. Vitamin D is a nutrient of concern among military personnel, and low serum vitamin D status has been associated with an increased risk of respiratory infection occurrence during basic training [20]. Recent evidence from British military recruits reported that only 21% had sufficient vitamin D status [serum 25(OH)D ≥ 50 nmol/L] during the winter months [20], and many military personnel begin deployment with suboptimal vitamin D status [63]. Among U.S. Marine Corps recruits undergoing initial military training (IMT), a positive association was observed between serum vitamin D status during times of high stress and salivary SIgA; additionally, vitamin D supplementation in this group was associated with increases in SIgA [64]. The potential utility of vitamin D supplementation is highlighted by data compiled over a six-year period from Air Force, Army, and Marine Corps trainees, indicating that roughly 30% of recruits are vitamin D deficient upon entering IMT [65]. Additional evidence from a randomized controlled trial supports that supplementation by simulated sunlight or oral D_3_ during basic training may reduce the severity and duration of infection [20].

Strong evidence indicates that iron nutriture is linked with infectious disease risk. The body’s iron stores support both innate and adaptive immunity [3,66], and iron’s vital role in oxygen transport comes into effect during activities that stress the aerobic energy system [67]. Assessment and treatment of iron status among premenopausal women and active populations who are at risk for depletion of iron stores is of key importance, as decrements in iron status additionally affect cognitive and physical function [68,69]. Importantly, studies indicate iron is a nutrient of concern among military personnel in certain environments, which contributes to their vulnerability to compromised immune function [18,70,71]. A recent cross-sectional study observed a decline in iron status among male recruits undergoing 15 months of IMT [72]. It is likely that during periods of stress, such as repeated/prolonged periods of strenuous exercise, inflammation potentiates the hepcidin-mediated decline in iron status [73,74], which in turn degrades immune function. For example, a randomized-controlled trial in male Army personnel showed that a simulated 72 h military operation involving energy deficit, sleep deprivation, and arduous physical training resulted in a greater inflammatory response (evidenced by increased C-reactive protein and interleukin-6), elevated hepcidin levels, and subsequent decreased iron absorption [75]. Thus, this preliminary evidence postulates that improving dietary iron intake may mediate favorable changes in immune competence during military training when an energy deficit is inevitable [76] and hypoferremia of inflammation occurs [74].

Vitamin C supports the growth and function of innate and adaptive immune cells, epithelial barrier integrity, phagocytosis, antibody production, and white blood cell migration to the infection site [66]. Moreover, vitamin C protects against reactive oxygen species and reactive nitrogen species during the oxidative burst that occurs when a phagocyte is activated by engulfing a pathogen [2,66]. Although vitamin C is not considered a nutrient of public health concern within the U.S. [77], data suggest rising trends in the prevalence of inadequate intake among the population [78]. Some evidence suggests that individuals who regularly perform repeated bouts of vigorous physical activity, such as athletes and military personnel, may require more vitamin C to limit exercise-induced immune depression and reduce infection risk compared to less active people [69]. Additionally, vitamin C enhances the bioavailability of iron absorption. 

Zinc has a role in the growth, development, and maintenance of innate and adaptive immune cells [2]. Zinc impacts the immune competence of the gut through effects on the gut mucosal immune system by minimizing parasite survival and driving changes in systemically dispersed immune responses [79,80]. Furthermore, diminished zinc status has been associated with increased susceptibility to viral infection and malarial parasitemia [79,81]. Although average reported daily intakes meet the Recommended Dietary Allowance (RDA) in the U.S. population and zinc deficiency is relatively low [82], it has been suggested that deficiency could develop in athletes in different training periods as a result of the oxidative stress from exercise, potentially related to zinc’s role in the antioxidant response through fluctuations in enzyme expression of copper zinc superoxide dismutase (CuZnSOD) [83]. Fluctuation in this enzyme expression stimulated by exercise is possibly linked to zinc fluctuation observed during exercise [83]. Zinc may also inhibit free radical production and increase antioxidant activity, all of which are essential roles in the antioxidant response to exercise [83]. Furthermore, as with iron, the inflammation experienced from strenuous exercise may contribute to an increase in circulating hepcidin and subsequent decreased zinc absorption [74]. Observational studies in humans [84,85,86] have shown that both exhaustive endurance and strength exercise modulate blood and urinary zinc levels through decreasing blood zinc levels while increasing urinary and sweat losses [83,87], which is additionally relevant to military personnel who regularly undergo strenuous training and occupational stress. However, accurate assessment of zinc status is difficult due to the tight regulation of zinc homeostasis, and blood zinc levels do not necessarily reflect dietary zinc intake [87]. Moreover, blood zinc concentration is not reflective of cellular zinc concentration [83]. Associations between intense physical activity and reduced markers of zinc status in Soldiers have been observed in several studies, as military personnel may endure circumstances of decreased zinc intake combined with increased requirements [79]. A common issue among deployed military personnel is gastrointestinal infection or enteric disease, contributing to diarrhea, and potential subsequent hospitalization and/or loss of duty days [79]. Not only is zinc deficiency associated with a higher risk of diarrhea, but diarrhea is associated with higher losses of zinc [79]. As the factors placing military personnel at a higher risk for contracting infection have been previously discussed and strong evidence supports zinc as an immunomodulator, this population should consume at least the daily RDA for zinc intake in the U.S. (11 mg for adult men, 8 mg for adult women) while not exceeding the daily Tolerable Upper Intake Level (UL; 40 mg) [50,88].

### 3.3. Obesity and Infectious Disease

Obesity (BMI ≥ 30 kg/m^2^) is characterized by excess energy intake relative to energy expenditure and the storage of excess body fat [45], and its accompanying immune impairments are relevant to military personnel. As of 2018, 49% of all active duty military personnel are classified as overweight [35]. Recently, the 2021 Department of Defense Health of the Force Report indicated that 22% of all active duty military personnel are considered obese, a prevalence that increased by 12% from the previous year and by 17% since 2017 [16]. Associations have shown a disproportionately higher number of healthcare encounters by active duty Soldiers classified as obese compared to those classified as normal or overweight [89]. Additionally, between January 2020 and August 2021, overweight and obesity were the most common pre-existing comorbidities in Service Members who were diagnosed with or had a probable case of COVID-19 [26]. 

Individuals with obesity exhibit chronic low-grade inflammation with higher levels of circulating inflammatory markers (i.e., C-reactive protein, tumor necrosis factor-alpha, interleukin-6), adipokines, and cytokines, all of which may impair the immune response and have negative effects on the lung parenchyma and bronchi [7,36,90]. For example, obesity is recognized as a variable predisposing individuals to severe H1N1 influenza pulmonary infection [90]. The chronic inflammatory state characteristic of obesity can promote viral entry into target cells [91]. Additionally, altered T-lymphocyte responses, a marker of immune function, are commonly observed [45]. Individuals with obesity often consume inadequate micronutrients (including those pertinent to immune competence), likely because of poor diet quality [36,92]. Therefore, the combination of a chronic inflammatory state due to excess body fat, with the insufficient intake of micronutrients important for immune health, may place individuals with obesity at greater infection risk compared to individuals without obesity (Figure 1).

There are myriad clinical implications of obesity on immune function. For example, obesity has been associated with a higher risk for hospitalization during the flu season, a worse outcome after infection with influenza A, and a greater risk for respiratory illness and severity of infection in children [36]. It has been hypothesized that the burden of additional adipose tissue present in obesity may augment the pro-inflammatory response to extensive viral infection, as in the case of COVID-19, such that adipose tissue serves as a reservoir for viral spreading, shedding, immune activation, and an amplified cytokine response [93]. In support of that hypothesis, a retrospective cohort study among symptomatic COVID-19 patients (<60 years old) observed that those with a BMI of 30–34 and ≥35 kg/m^2^ were OR [95% CI] 1.8 times [1.2–2.7] and 3.6 times [2.6–5.3] more likely, respectively, to be admitted to the intensive care unit (ICU) compared to those with a lower BMI (<30 kg/m^2^) [94]. Furthermore, pooled data from a meta-analysis showed that not only did obesity increase the likelihood of unfavorable outcomes from COVID-19, but patients with obesity had a 48% increase in death [95]. Moreover, the clinical care burden of COVID-19 patients with obesity in the hospital can worsen the prognoses by complicating intubation procedures or positioning of the body [95].

Obesity has also been associated with decreased vaccine responsiveness. A meta-analysis revealed a relationship between obesity and an increased risk for non-responsiveness to the Hepatitis B vaccine, compared to non-obese persons (adjusted pooled OR [95% CI]: 2.46 [1.5–4.03]) [96]. More recently, a prospective observational study found that central adiposity was inversely correlated with the serological response to the anti-SARS-CoV-2 antibody titer following vaccination [97]. The inflammation resulting from excess adipose tissue can affect the cellular immune response to influenza vaccination, reducing the activation, maintenance, and activity of memory T cells [98]. Other potential mechanisms for decreased vaccine responsiveness associated with obesity include leptin-induced systemic inflammation, perturbation in adiponectin levels, B cell intrinsic inflammation, and suboptimal macrophage function and maturation [99,100]. Additionally, differences in viral shedding between normal-weight individuals and those with obesity have been observed, wherein viral shedding has been prolonged in adults with obesity. Among adults infected with symptomatic and paucisymptomatic/asymptomatic influenza A virus, viral shedding was prolonged by 42% and 104%, respectively, in those with obesity compared to those classified without obesity [101]. Furthermore, greater amounts of subcutaneous tissue present in overweight and obesity may affect vaccine efficacy and safety, as intramuscular compared to subcutaneous injections have resulted in fewer localized reactions [98].

## 4. Limitations

Due to the limited number of studies examining immune outcomes and factors affecting micronutrient status in the military population, several studies included in this review were published more than a decade ago. However, their relevance and associated findings are crucial in understanding the impact of military-relevant stressors and therefore necessitate inclusion.

## 5. Conclusions

Nutrition status and dietary intake influence the function and maintenance of the innate and adaptive immune systems. Optimal energy, protein, and micronutrient consumption is key, as both under- and over-nutrition are associated with adverse health outcomes and infectious disease risk in military populations. Limited evidence suggests immune-enhancing micronutrients of concern among military personnel in austere environments and undergoing strenuous training include iron, zinc, vitamin D, and vitamin C. Military working environments and occupational demands put military personnel at high risk for contracting infection, while at the same time compromising nutritional status by constraining adequate dietary intake in certain scenarios. Therefore, future research to further characterize the relationship between infectious diseases, immune function, and nutritional status in the context of the military setting and related stressors is necessary. Such studies are in the planning phase, and the results will be used to inform strategies for keeping military personnel healthy and reducing lost training days and healthcare costs. Additionally, obesity among military personnel is prevalent, so the impact of body weight and nutritional status on warfighter immune health and susceptibility to infectious disease warrants additional study. Such efforts will better inform policy and interventions that mitigate illness risk and infection transmission. Considering nutritional status and prioritizing efforts to optimize nutrient intake is one approach for reducing disease burden and improving readiness. 

## Data Availability

No new data were created or analyzed in this work. Data sharing is not applicable to this article.
